# Genome sequence of *Staphylococcus nepalensis* ZZ-2023a, isolated from *Nasonia vitripennis*

**DOI:** 10.1128/mra.00802-23

**Published:** 2023-12-12

**Authors:** Zhengyu Zhu, Runbiao Wu, Guan-Hong Wang

**Affiliations:** 1 State Key Laboratory of Integrated Management of Pest Insects and Rodents, Institute of Zoology, Chinese Academy of Sciences, Beijing, China; 2 College of Life Sciences, Hebei University, Baoding, China; University of Delaware College of Engineering, Newark, Delaware, USA

**Keywords:** *Staphylococcus nepalensis*, *Nasonia vitripennis*

## Abstract

We isolated a strain of *Staphylococcus nepalensis* from *Nasonia vitripennis* and presented the draft genome sequence of this strain. This research was conducted at the Institute of Zoology, Chinese Academy of Sciences (Beijing, China). The genome spans 2,910,033 bp, distributed over 144 contigs, with a G+C content of 33.33%.

## ANNOUNCEMENT

*Staphylococcus nepalensis* is a Gram-positive coccus that was originally discovered in the respiratory tract of goats in 2003 (1). This bacterium was later detected in various insects, food, and human urine (2 – 4). We sequenced the genome of *S. nepalensis* isolated from *Nasonia vitripennis. N. vitripennis* has evolved into a well-established research model, owing to its distinctive biological attributes, particularly in the domains of parasitism, epigenetics, evolutionary genetics, and host-microbe interactions (5, 6). However, it is not yet clear whether it is pathogenic or not. Nevertheless, the contribution of *Staphylococcus* species, especially *S. nepalensis*, to the growth and development of wasp hosts has not been elucidated and needs further investigation.

*S. nepalensis* ZZ-2023a was isolated and identified from *N. vitripennis* at the Institute of Zoology, Chinese Academy of Sciences (Beijing, China). Adult sample was collected and washed with 1 mL 70% ethanol solution, followed by 1 mL 10% bleach solution for 2 min, and then washed with 1 mL sterile water three times. The sample were ground in sterile phosphate-buffered saline, homogenized, and then diluted before being plated on Individual bacterial colonies were obtained by streaking on Luria-Bertani (LB) agar plates, followed by liquid LB culture at 35°C–37°C for single-bacterium enrichment (2). The taxonomic identity was confirmed by amplifying the partial 16S rRNA sequence using the universal bacterial primers 27F and 1492R.

The extracted bacterial genomic DNA using the TIANamp Genomic DNA Kit (Tiangen, China) was sent to Biomarker technologies (Beijing, China) for sequencing under sequencing platform of Illumina NovaSeq 6000, insert size 350 bp, paired-end 150 bp read lengths and sequencing depth of more than 100×. The high-throughput sequencing data were evaluated using FastQC v0.11.5 (with default parameter) (7). In order to remove the adapter and low-quality reads, Quality control was performed using fastp v0.23.2 (with parameters -z 4 -q 20 u 30 n 10) (8). Around 9.16 M high quality reads were retained and assembled using SPAdes v3.15.4 (with parameters -k 21,33,55,77 --careful) (9). The quality of genome assembly was evaluated using QUAST v5.2.0 (with default parameter) (10). We eventually obtained 144 contigs with approximately 467-fold coverage. The *N*_50_ and *L*_50_ values were 488,258 bp and three contigs, respectively. The genome of *S. nepalensis* ZZ-2023a consists of 2,910,033 nucleotide, and G + C content was 33.33%. We also evaluated the number and length of assembled Scaffold using QUAST v5.2.0 (with default parameter), which were 138 scaffolds and 2,910,553 bp, respectively. Only contigs longer than 1,000 bp were used for gene annotation which was performed using NCBI Prokaryotic Genome Annotation Pipeline (11), and the genome encodes 2,981 protein-coding genes, 7 rRNA genes, and 56 tRNA genes. We present the genome sequence of *S. nepalensis* ZZ-2023a isolated from the wasp *N. vitripennis*, providing valuable information and a research foundation to enhance our understanding of microbial regulation mechanisms.

## Data Availability

All data reported in this paper have been deposited in the National Center for Biotechnology Information, and the Illumina sequencing raw data can be accessed via BioProject ID PRJNA967228, BioSample ID SAMN34152378, or Sequence Read Archive (SRA) accession number SRR24442193. The 16S rRNA gene amplicon sequence and assembled genome sequence were also deposited in GenBank with accession numbers OQ927070 and JASGWV000000000, respectively.
